# The Impact of Electronic Health Records on Family Physicians During Simulated Virtual Encounters: Exploratory Mixed Methods Study

**DOI:** 10.2196/84916

**Published:** 2026-05-19

**Authors:** Lisa Shah, Helen Monkman, Andre Kushniruk, Kendall Noel, Douglas Archibald, Farhad Motamedi, Gary Viner

**Affiliations:** 1 School of Health Information Science, Faculty of Health, University of Victoria, Human and Social Development Building room A202, 3800 Finnerty Road (Ring Road), Victoria, BC, V8P 5C2, Canada, 1 250-721-8575, 1 250-472-4751; 2 Department of Family Medicine, Faculty of Medicine, University of Ottawa, Ottawa, ON, Canada

**Keywords:** electronic health records, electronic medical records, telemedicine, patient-centered care, medical education, patient simulation, cognitive load

## Abstract

**Background:**

This exploratory study investigated the impact of computer use on physician performance during clinical simulations. Standardized patient (SP) scenarios used in family practice certification examinations were adapted to include the use of the electronic health record (EHR).

**Objective:**

The goal was to compare the impact of EHR use during simulated virtual patient encounters on resident physicians’ and staff physicians’ patient-centeredness (PC) and overall clinical performance, as well as to measure the cognitive load (CL) imposed by EHR use.

**Methods:**

Sixteen participants each completed 2 video telemedicine simulations with SPs. One simulation case included limited past medical history for the SP in the EHR, while the other did not. Participants were instructed to completely document the encounter using the EHR. Participants’ self-perceived CL was measured using the raw National Aeronautics and Space Administration Task Load Index (NASA-TLX). Video recordings were analyzed for participant PC and overall clinical performance. In addition to interacting with the EHR, multiple participants also conducted internet searches. The proportion of time that participants spent interacting with the computer, either using the EHR or searching the internet, was calculated. Inductive qualitative coding of a subset of video recordings (18 of 32 encounters) was performed, with a focus on signs of stress/CL. All videos were assessed for usability problems.

**Results:**

Staff physicians (n=6) scored higher on PC compared to resident physicians (n=10) for both cases, though differences were not statistically significant after correction for multiple comparisons (family-wise error rate). Physicians’ overall CL, as measured by the raw NASA-TLX, was not significantly correlated with computer use. Exploratory qualitative data analysis found both verbal and nonverbal signs of stress/CL due to computer use while interacting with the SPs. The proportion of time displaying nonverbal signs of stress/CL was calculated for a subset of participants (6 resident physicians and 3 staff physicians). Participant interpretations of instructions to completely document the encounter using the EHR varied widely. It is likely that participants’ usual style of documenting, either primarily during or after patient encounters, impacted their use of the EHR while SPs were present.

**Conclusions:**

Use of the computer during video telemedicine appointments may negatively impact physician PC and overall clinical performance. Exploratory qualitative coding identified both verbal and nonverbal signs of stress/CL when participants interacted with the computer and the patient simultaneously. Increased clinical experience helped to mitigate the negative impact of computer use. If the use of the EHR is included in physician certification examinations, clear instructions regarding which tasks must be completed in the EHR during interactions with SPs should be provided.

## Introduction

### Background

Electronic health records (EHRs) have become ubiquitous in both hospital and office environments. Primary care physicians, on average, spend more time interacting with the EHR than on direct patient care [1-3]. In 2024, 95% of Canadian physicians used an EHR to document patient encounters [4]. In most countries, the term EHR is now used to refer to both office-based and hospital-based record systems, which create electronic versions of traditional paper charts.

Interaction with the EHR may impact physician-patient communication, physician clinical reasoning, and patient satisfaction [5-7]. Patient-centered communication may decrease, and pauses in conversation may increase when physicians spend more time looking at a computer screen [6]. Despite this well-known impact of the EHR on patient care, training on the effective use of the EHR has not been added to medical school curricula in Canada [8,9]. The 2025 Canadian Medical Student Digital Health Survey had an 8.3% (1103/13,217) response rate [10]. This survey found that 57% (630/1103) of responding medical students did not receive formal digital health training in medical schools [10]. Confidence in performing tasks related to the EHR ranged from 8% (92/1103) for using an eReferral system to a high of 31% (339/1103) for documenting an encounter [10]. The largest barrier to learning about digital health, cited by 65% (713/1103) of medical students, was a lack of formal instruction [10]. Most physicians in Canada learn to use EHRs while charting patient encounters during clinical placements in medical school and residency.

The documentation burden related to EHRs has added to physicians’ workloads and is contributing to physicians’ burnout in Canada [11,12]. The increase in telemedicine during the COVID-19 pandemic has resulted in an increase in the time spent by ambulatory care physicians working in the EHR [13]. Charting contemporaneously during patient visits can decrease the time physicians need to spend working in the EHR after clinic hours. Prompt documentation can also improve the accuracy of medical records. However, the negative impacts on patients due to physicians’ distraction while interacting with the EHR during patient appointments must be avoided when possible.

### Goals of This Study and Hypotheses

This study sought to examine patient-centeredness (PC) during 2 standardized clinical simulations typical of new patient family medicine visits. We hypothesized that experienced family physicians with more years spent refining communication skills would be more patient-centered compared to resident physicians. Another premise was that interaction with the EHR while the standardized patient (SP) was present would impact resident physicians more than staff physicians, who were assumed to be more confident in the use of the EHR and more aware of how to remain patient-centered during patient visits involving the use of the EHR. A simulation case containing historical medical information in the EHR and thus requiring participants to navigate the EHR to find this information was hypothesized to create a higher cognitive load (CL) for participants compared to a simulation case without any medical history.

This project was originally planned for in-person interactions between physicians and SPs to assess clinical reasoning and the impact of a newly implemented EHR. Due to the COVID-19 pandemic, the format was changed to a virtual environment, with physicians interviewing SPs over Zoom. The focus became restricted to PC, a component of clinical reasoning, and overall clinical performance scores as assessed by physicians trained in certification examination scoring.

Virtual encounters increased during the COVID-19 pandemic, and this shift in appointment format has persisted in Canada to a lesser extent in the following years [14]. Assessing the impacts of EHR use during virtual encounters is thus an important area of study. In a survey of patients’ feelings about video telemedicine visits, some patients expressed concerns that their physicians paid less attention to them and that it was difficult to find opportunities to speak up during video telemedicine visits [15]. Therefore, this study’s pivot to a video telemedicine format provided an opportunity to study the impact of a virtual format on physician-patient communication.

### Prior Work

#### Patient-Centered Clinical Method

The patient-centered clinical method was developed in the 1980s as a conceptual framework and was subsequently revised several times [16]. It is based on a 4-component framework: (1) exploring the patient’s illness experience, (2) trying to understand the whole person, (3) finding common ground, and (4) improving the patient-physician relationship [16,17]. The Patient Perception of Patient-Centeredness (PPPC) questionnaire was developed in the 1980s and has been used as a survey tool for patients in family practice populations and with SPs [16]. The PPPC was revised in 2019, with the new version termed the revised PPPC (PPPC-R). This version contains 18 items, with possible responses to each item ranging from 1 (*completely*) to 4 (*not at all*). Given this reverse anchoring in the PPPC-R, a lower score indicates a higher PC rating.

The College of Family Physicians of Canada (CFPC) developed an instrument to measure resident physicians’ PC and overall clinical performance for their certification examination. This is termed the simulated office oral (SOO) examination and is scored by experienced family physicians using a standardized rubric [17]. A comparison between the PC score from the SOO and the PPPC-R measure of PC has not been explicitly described in past studies. We note that a higher score indicates better performance in terms of PC on the SOO, whereas a lower score indicates better performance on the PPPC-R. The National Library of Medicine Medical Subject Headings, a controlled vocabulary thesaurus for PubMed, uses the spelling *patient-centered* [18]. Therefore, the spelling *patient-centered* will be used throughout this paper.

#### Cognitive Load

In addition to investigating the impact of the EHR on physician communication, this study also sought to measure the cognitive impact of the EHR on physician participants. Psychometric instruments measure self-perceived CL. The National Aeronautics and Space Administration Task Load Index (NASA-TLX) was the most frequently used psychometric measure of CL found in a literature review of CL measurement related to EHRs [19]. Therefore, this tool was chosen to measure the cognitive impact of the EHR on physicians.

The NASA-TLX contains 6 domains of task load: mental demand, temporal demand, physical demand, frustration, performance, and effort [20]. The traditional method of scoring the NASA-TLX asks participants to rate each domain on a scale from 0 to 100 and then compare pairs of domains to weight them in importance [21,22]. The weighting, combined with the Likert scale scoring of each domain, is used to obtain an overall score for each participant. The traditional method of scoring the NASA-TLX has fallen out of favor in recent years, as it can be time-consuming to collect the required data from participants, and the calculation of overall workload is more complicated [23,24].

The simplified raw NASA-TLX scoring method uses the unweighted mean of the subscale scores, which minimizes the time required for participants to complete the questionnaire [25,26]. Raw NASA-TLX scores have been used in studies of clinicians’ workload [27,28]. Currently, there is no consistent method for scoring the NASA-TLX in studies of health care provider workload. Both the traditional and raw methods for calculating the overall workload from the 6 dimensions of the NASA-TLX can be problematic, and thus, the use of the 6 dimensions separately may be preferred [23].

#### Verbal and Nonverbal Signs of Stress/CL

Prior studies have found both verbal and nonverbal indicators of stress/CL during human-computer interaction. Multiple studies have noted spontaneous self-touch gestures to be signs of stress, anxiety, or CL, though the neurophysiological mechanism is still not well understood [29]. Other terms for these gestures include self-adaptors, body-focused movements, displacement behaviors, and self-directed behaviors. These behaviors may help regulate negative emotional states and increase focus during cognitively demanding tasks [29-32]. Spontaneous self-touch or self-directed behaviors have been defined as involuntary hand movements involving touching, rubbing, grooming, or scratching one’s body, as well as fumbling with adornments, such as clothing or accessories [32,33].

Grunwald et al [30] discovered that spontaneous facial self-touch gestures increased dramatically during a delayed memory task designed to be emotionally and cognitively straining by playing unpleasant sounds. The facial self-touches were found to help with working memory maintenance and emotional regulation. Spille et al [34] found that spontaneous facial self-touches were associated with neurophysiological changes related to the regulation of attention and emotion. Spontaneous self-touch of the face increased in frequency when participants’ attention was distracted by unpleasant sounds during a delayed memory task.

Previous linguistics research has reported that when a speaker is performing a task under stressful conditions, such as high cognitive workload or psychological tension, speech features may change. These changes include observable and measurable speech features, which as a group are termed disfluencies; they include stuttering, repeating words, pauses between utterances, fillers, and repairs [35,36]. Fillers, also known as filled pauses, include vocalizations such as *uh*, *uhm*, and *mh*, as well as words such as *well*, *you know*, and *yeah* [36-38]. Repairs occur when the person speaking stops what they were saying and restarts their sentence. Speech hesitations, which include silences and fillers, have been found to increase in frequency due to high CL and stress [38,39]. A recently published scoping review reported that an increase in speech disfluencies under dual-task conditions that imposed high CL was identified in 5 out of 6 studies that met inclusion criteria [40].

### Objectives

This exploratory study aimed to determine if the use of the EHR during SP encounters had an impact on physicians’ PC and clinical performance and whether the impact varied between residents and staff physicians. Physician examiner–assigned PC scores were compared to PPPC-R questionnaire scores to assess if there was a difference between physician and patient perceptions of participant PC. Physicians’ CL was measured using the raw NASA-TLX to determine if use of the EHR and internet searches while interacting with SPs impacted physician self-perceived CL. Inductive qualitative analysis was also carried out to assess usability problems and signs of stress/CL. Possible correlations between computer use and physicians’ CL, PC, and overall clinical performance scores were investigated.

## Methods

### Ethical Considerations

The ethical approval for this study was granted by the Ottawa Health Science Network Research Ethics Board (OHSN-REB number 20190160‐01H), which complies with the requirements of the Tri-Council Policy Statement: Ethical Conduct for Research Involving Humans (TCPS2) [41]. All participants were given a participant informed consent form to review prior to deciding whether to participate. Identifying information of participants was kept confidential by the research team. Research data files were stored in a secure SharePoint (Microsoft) site managed by The Ottawa Hospital, which only members of the research team could access. Login required 2-factor authentication. Participants were assigned ID numbers. Participant scores on each simulated case were only recorded with participant ID numbers, not with participant names. SPs and simulated clinical scenarios were used to alleviate privacy concerns. Participants were compensated for their time with a CAD $100 gift card.

### Recruitment

We aimed to recruit 15 first-year residents from the Family Medicine program at the University of Ottawa and 15 experienced academic family physicians with more than 5 years of practice, based at 2 campuses. This represented a convenience sample of first-year residents and staff physicians in the Family Medicine department using the same EHR, which was implemented in June 2019. The participants were invited by email to participate in this study. First-year residents were selected as they would have the least experience with EHRs and PC. Due to an insufficient number of first-year resident volunteers, we also invited early second-year residents to enhance our participant pool. We believed that early second-year residents would also perform differently from the experienced physicians, as they have had fewer years of practice with patient-centered communication while using the EHR. Recruitment was a challenge, given the timing of the study soon after the onset of the COVID-19 pandemic. The stress of working in the pandemic environment, combined with the academic and teaching demands of the residents and staff physicians, limited the number of residents who agreed to participate to 10 and staff physicians to 6. Participants filled out a demographic questionnaire, which included information on age group, gender, decade of graduation from medical school, and number of years in practice.

### Study Design

SPs, played by actors, were used in simulated clinical scenarios to negate the variability of actual patient encounters and alleviate privacy concerns. Two actors were trained by experienced physician examiners with support from the Ottawa Exam Center prior to the COVID-19 pandemic. There was no opportunity to retrain them or readily monitor their performance once this study had to pivot to a virtual format. Two clinical simulations drawn from the CFPC certification examination in family medicine were adapted for use in this study. The CFPC SOO exams do not include the use of the EHR. Therefore, the cases were slightly modified to include documentation in the EHR. Past medical history in the EHR was included for 1 SP case (abbreviated as *PMHx* case), including a consultation note from a visit to an emergency department, a computed tomography scan report, and an X-ray report. The other case, referred to as the *NoHx* case, did not include any past medical information in the EHR.

**Figure 1.**
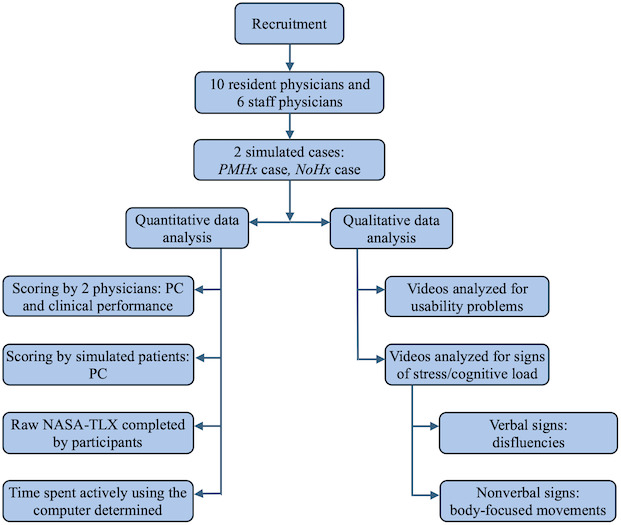
Study flow diagram. NASA-TLX: National Aeronautics and Space Administration Task Load Index; *NoHx* case: simulation case that did not include any past medical history in the electronic health record (EHR); PC: patient-centeredness; *PMHx* case: simulation case that included some past medical history in the EHR.

The encounters were designed to simulate routine new patient family medicine visits. Participants were provided with the following instructions on EHR use: “You will be expected to completely document the encounter, using our electronic health record. You will have about 20 minutes to interact with the patient and an additional 5 minutes to complete your documentation of the encounter.” Participants were also advised that documentation would occur in the EHR training environment, where EHR customizations, templates, and smart phrases were not available. The full instructions given to participants are detailed in Multimedia Appendix 1. Time limits were extended by 5 minutes from the standard 15-minute SOO exam format to 20 minutes with the SP, plus an additional 5 minutes allotted for documentation after the encounter. Figure 1 summarizes the quantitative and qualitative assessment methods used in this study.

Encounters were completed virtually between 2021 and 2022 to accommodate physical distancing requirements and the continuing challenges of a post-COVID-19 clinical environment. The video telemedicine simulated encounters were recorded using Zoom. Ten resident physicians and 6 staff physicians from an academic, university-affiliated family practice clinic each completed 2 sequential SP scenarios (both the *PMHx* case and the *NoHx* case) during one session, with a research assistant monitoring the Zoom encounters remotely.

After each scenario, participants completed the raw NASA-TLX questionnaire. The raw NASA-TLX score was used during data analysis, as well as separate scores on single dimensions of the NASA-TLX that were determined by group consensus to be the most relevant (mental demand, time pressure, and frustration). The SPs answered a survey regarding their perception of the participants’ PC, using the PPPC-R questionnaire.

Physicians’ PC and overall clinical performance were rated based on the scoring standards of the CFPC SOO exam. Two family physicians experienced in scoring these exams evaluated each participant’s PC and overall clinical performance.

Transcripts of the audio generated by Zoom were manually corrected when speaker identification or words spoken were captured incorrectly. Transcripts and timestamps from the Zoom video recordings were analyzed to calculate the time spent actively using the EHR, as well as the time spent conducting internet searches. Active EHR time was counted until there were no keystrokes or mouse activity for the following 5 seconds. This method was used by the EHR vendor to determine active EHR use time and was selected for this study to make the count as objective as possible [1].

Video recordings were analyzed to determine the number of EHR pages, also known as screens, that participants clicked on during their interactions with the SPs. The EHR navigation count was calculated as the number of times participants switched between different pages of the EHR during the time they were interacting with the SPs. Participants were given 5 minutes after the SPs logged off to complete tasks in the EHR. Any tasks completed using the EHR after the SPs logged off were not included in the analysis, as they were not relevant to interactions with the SPs.

Exploratory qualitative analysis by a single coder was based on a video-coding framework developed for investigating cognition and usability in health care informatics [42]. Coding was discussed during group meetings and refined based on an inductive approach. A codebook was developed, and transcripts were coded and analyzed using NVivo software version 14 (Lumivero). The initial focus was on usability codes. As the analysis of the video recordings continued, signs of stress/CL became evident through both verbal and nonverbal behaviors, and thus, new codes were developed. The analysis of video recordings included the calculation of the proportion of time displaying spontaneous self-touch/self-directed behavior for a subset of 6 resident physicians and 3 staff physicians, for a total of 18 encounters. This was a very time-consuming task, and thus, it was impractical to code all 32 encounters for nonverbal signs of stress/CL. Spontaneous self-touch/self-directed behavior frequency was calculated as a proportion of total time while the SPs were present [32].

## Results

### Principal Results

Sixteen participants each completed the 2 SP simulation cases and the raw NASA-TLX questionnaire after finishing each encounter. Table 1 details participant demographics.

Data were graphically summarized using box plots created in RStudio (version 2025.09.2+418; Posit Software, PBC), which display the 25th, 50th, and 75th percentiles and outliers. Medians and IQR values of variables by the physicians’ experience level and simulation case are detailed in Table 2.

**Table 1.** Demographic data (N=16).

Characteristics	Values, n (%)
Gender	
Male	6 (37.5)
Female	10 (62.5)
Professional role	
Resident physician	10 (62.5)
Staff physician	6 (37.5)
Age (y)	
21‐30	5 (31.2)
31‐40	7 (43.8)
>40	4 (25)

**Table 2.** Median (IQR) values of variables by the physicians’ experience level and case (N=16).

Variables	Residents *PMHx*^a^ case (n=10), median (IQR)	Staff *PMHx* case (n=6), median (IQR)	Residents *NoHx*^b^ case (n=10), median (IQR)	Staff *NoHx* case (n=6), median (IQR)
Computer use				
Time proportion	0.42 (0.32‐0.47)	0.38 (0.32‐0.50)	0.30 (0.14-0.36)	0.30 (0.263‐0.34)
Page count	3.0 (2.0‐5.0)	2.5 (1.2‐3.8)	1.0 (1.0‐2.0)	1.5 (1.0‐2.8)
Navigation count	9.5 (4.2‐26.0)	11.0 (2.5‐20.2)	1.0 (1.0‐3.5)	1.5 (1.0‐6.5)
Physicians’ performance				
Patient-centeredness score	6.0 (4.0‐8.0)	12.5 (9.0‐16.0)	16.5 (12.0‐17.8)	21.0 (18.5‐22.8)
SOO^c^ exam total score	20.0 (13.0‐24.8)	27.0 (18.5‐34.8)	32.0 (27.0‐34.5)	44.0 (41.5‐46.5)
PPPC-R^d^	3.2 (2.9‐3.5)	1.8 (1.7‐2.4)	1.8 (1.6‐2.4)	1.4 (1.2‐1.8)
Cognitive load (NASA-TLX^e^)				
Raw NASA-TLX mean	46.3 (40.2‐53.0)	44.2 (36.5‐53.5)	54.0 (47.2‐57.2)	39.9 (32.7‐50.9)
NASA frustration	54.5 (21.5‐70.5)	44.0 (12.2‐60.0)	54.5 (38.8‐69.2)	15.5 (8.5‐52.5)
NASA mental demand	65.0 (50.8‐74.0)	69.0 (53.5‐79.2)	72.5 (60.2‐78.8)	64.5 (39.5‐81.2)
NASA time pressure	37.5 (31.2‐67.2)	49.0 (46.5‐66.5)	55.5 (19.0‐76.0)	57.5 (55.0‐60.0)

a *PMHx* case: simulation case that included some past medical history in the EHR.

b *NoHx* case: simulation case that did not include any past medical history in the EHR.

c SOO: simulated office oral.

d PPPC-R: Revised Patient Perception of Patient-Centeredness (PPPC) questionnaire. On the PPPC-R scale, a lower score indicates better performance due to reverse anchoring.

e NASA-TLX: National Aeronautics and Space Administration Task Load Index.

Due to small and unequal group sizes and uncertainty regarding the normality of the data, nonparametric statistical tests conducted in RStudio were used for data analysis [43,44]. The predetermined α level was .05. Due to multiple comparisons between groups, the Holm-Bonferroni correction test was applied to reduce the likelihood of false-positive findings (ie, to control for the family-wise error rate). The Wilcoxon-Mann-Whitney rank sum test was used to compare resident physicians to staff physicians on the outcome variables of computer use, PC scores, SOO total scores, and NASA-TLX scores. Complete Wilcoxon Mann-Whitney rank sum test results and thresholds as per the Holm-Bonferroni correction test are provided in Tables S1 and S2 in Multimedia Appendix 2. Not surprisingly, given the small sample size, none of the observed trends achieved statistical significance.

**Figure 2.**
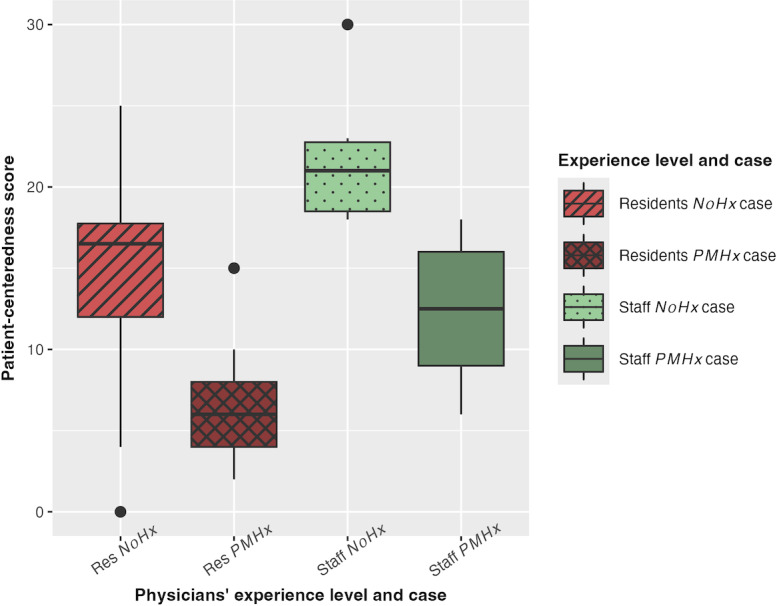
Patient-centeredness score by the physicians’ experience level and case. EHR: electronic health record; *NoHx* case: simulation case that did not include any past medical history in the EHR; *PMHx* case: simulation case that included some past medical history in the EHR; Res: resident physicians; Staff: staff physicians.

As hypothesized, staff physicians received higher PC scores (as rated by physician examiners) compared to resident physicians, though differences between groups were not statistically significant after correction for the family-wise error rate. Figure 2 displays the median PC score for each group of physicians by the experience level and the simulation case, as well as the first quartile (25th percentile), third quartile (75th percentile), and outliers shown as points outside the box plots.

SP ratings of participants’ PC using the PPPC-R questionnaire also found that staff physicians scored better than resident physicians for both the *PMHx* case and the *NoHx* case. Due to reverse anchoring, lower scores on the PPPC-R scale indicate better performance, as detailed in Table 2.

The use of the EHR and internet searches during SP appointments varied widely. The majority of EHR usage time was spent in the “Progress Notes” section. During appointments with SPs, EHR page counts varied from 0 to 11, while EHR navigation counts varied from 0 to 123. Participants clicked on only the “Progress Notes” section of the EHR in 11 of 32 encounters. Ten out of 16 participants attempted to view past medical history reports for the *PMHx* case. Some resident physicians had difficulty finding imaging order forms, as well as locating past imaging reports scanned into the EHR, which led to an increase in navigation count for these residents. Staff physicians were familiar with the location of scanned documents in the EHR and did not encounter this difficulty. Resident physicians conducted more internet searches during appointments with SPs than staff physicians. Four residents searched the internet during 6 encounters (2 during both encounters they completed) versus 1 staff physician during 1 encounter.

Most participants did not attempt to order investigations or complete referrals during SP appointments. Participants who completed more EHR tasks while the SPs were present had lower performance scores, though this finding was not statistically significant after correction for the family-wise error rate. Participants who attempted to input orders or complete EHR *Wrap Up* tasks (such as booking follow-up appointments), while the SPs were present, received the lowest PC scores in their experience-level groups (residents or staff physicians). Some participants did not hear time prompts over Zoom, resulting in appointment times ranging from 10.35 to 33.71 minutes. Due to this issue, active time spent using the computer was calculated as a proportion of overall appointment time.

The proportion of time spent interacting with the EHR varied by SP case, as was anticipated due to the inclusion of historical medical information in the EHR for only the *PMHx* case. Time spent using the computer was not significantly different between resident physicians and staff physicians for either SP case, though both groups spent more time interacting with the EHR during the *PMHx* case, which contained historical medical information in the EHR.

Correlations between computer use and other variables were assessed using the nonparametric test Kendall τ_b_ in RStudio [45,46]. With the Holm-Bonferroni correction method applied to control for the family-wise type 1 error rate, the results were not statistically significant. The complete results of Kendall τ_b_ tests of correlation and thresholds, as per the Holm-Bonferroni method, are provided in Tables S3-S6 in Multimedia Appendix 2.

### Qualitative Results

The exploratory qualitative analysis of video recordings found an increase in signs of stress/CL when participants were using the computer while the SPs were present. Verbal signs of stress/CL included the use of filler words, repetition of words, restarting sentences, stuttering, nonlexical conversational sounds, and pauses lasting longer than 5 seconds during conversations with SPs. Nonlexical conversational sounds include verbalizations, such as “mm-hm” and “uh-uh” [47]. Filler words include words such as “okay, sure, yeah, like, all right, so, and perfect,” which were repeated by participants when they were multitasking by speaking to the SP and using the EHR concurrently. Due to the frequency of verbal signs of stress/CL, it was impractical to calculate counts to compare residents and staff physicians.

Participants sometimes missed information stated by the SPs when they were distracted by interactions with the computer. For example, several resident physicians asked the SP to repeat details regarding symptoms that the patient had already stated while the resident was interacting with the EHR. One resident physician also asked the SP if she was married, even though she had already mentioned her spouse multiple times. Verbal turn-taking was challenging in the video telemedicine setting. Accidental interruptions seemed to occur more often compared to in-person conversations. This problem has been noted in studies examining video telemedicine interactions, as there are short periods of latency in video-mediated interactions that can disrupt conversational turn-taking [48].

The proportion of time spent displaying nonverbal signs of stress/CL was calculated for a subset of participants (2 encounters each for 6 residents and 3 staff physicians, for a total of 18 encounters). The nonverbal signs of stress included self-directed touch of the face, head, neck, or hands, as well as adjustments to adornments, such as glasses. During the PMHx case, the median proportion of time spent displaying nonverbal signs of stress/CL while the SP was present was 0.16 (0.05-0.24) among resident physicians compared with 0.06 (0.04-0.10) among staff physicians. During the NoHx case, the median proportion of time spent displaying nonverbal signs of stress/CL was 0.10 (0.02-0.14) among resident physicians compared with 0.05 (0.04-0.06) among staff physicians.

Sample sizes included in the calculation of the proportion of time spent displaying nonverbal signs of stress/CL (n=6 for resident physicians and n=3 for staff physicians, for a total of 18 encounters) were too small to conduct statistical analysis for possible associations between variables.

## Discussion

### Principal Results

This study investigated the use of the EHR and internet searches by family practice resident physicians and staff physicians during simulated video telemedicine appointments with SPs. Staff physicians had higher PC and overall clinical performance scores compared to resident physicians, as was hypothesized due to their increased experience in remaining patient-centered while interacting with the EHR during patient appointments. Differences in performance scores based on the physicians’ experience level were not statistically significant after correction for the family-wise error rate, possibly due to small sample sizes and resulting low power. The active use of the computer, including both interacting with the EHR and conducting internet searches, had a negative impact on physicians’ PC. This finding was evident through the qualitative video analysis, though correlations between the proportion of time spent using the computer while interacting with SPs and physician performance scores did not achieve statistical significance after correction for the family-wise error rate.

There was a wide variation in the tasks participants chose to complete in the EHR while the SPs were present. While this partly reflects a major limitation of this study due to a lack of specificity in the instructions, it is also likely related to the personal documentation styles of the residents and staff physicians who participated in this study. Some physicians prefer to document in the EHR while speaking with patients, while others take notes on paper and complete documentation in the EHR after the encounter has ended. Many physicians adopt an approach somewhere between these 2 strategies, with documentation and ordering tasks in the EHR completed both during and after patient encounters. Therefore, the finding that some participants completed more documentation and other EHR tasks while the SPs were present is unlikely to be solely due to their interpretations of the instructions.

If a similar study is conducted in the future, clearer instructions for participants are recommended, including whether participants should order relevant investigations and referrals in the EHR. Participants who interpret the instructions as requiring them to order laboratory investigations, imaging, and referrals while the SP is present are disadvantaged in terms of their overall clinical performance and PC scores. Screen-recording software could be used to capture keyboard and mouse activity, thus simplifying the calculation of time spent actively using the EHR or searching the internet. Improved SP training and feedback are recommended to achieve consistency in acting and the provision of medical details when questioned by participants. The selection of case scenarios where interpretation of body language is key should be avoided, as this can be missed over Zoom.

### Comparison With Prior Work

This study’s findings align with those of Street et al [6], who concluded that medical school curricula should be modified to include EHR management skills. When physicians focus too much on the EHR, this can decrease patient-centered communication and cause long conversational pauses. Physicians in training should be taught to maintain conversational flow with patients while interacting with the EHR [7].

Currently, there is a lack of formal teaching for medical learners on effective communication during video telemedicine consultations [10]. Given the increase in telemedicine that occurred during the COVID-19 pandemic and has persisted to some extent in Canada, consideration should be given to teaching medical students and resident physicians interpersonal skills specific to telemedicine. Important skills for video telemedicine visits include explaining the process to patients, using clear language, maintaining active listening, and directing eye contact toward the camera [49]. Tone of voice and nonverbal communication are also important; physicians should face the camera and minimize gaze directed at the keyboard. When entering notes into the EHR, narrating the purpose of doing so to the patient can be helpful [50]. Lighting and positioning of the video telemedicine platform within the computer screen should be considered to ensure that both the physician’s and patient’s faces are clearly visible [50]. Distracting background noise should be kept to a minimum, and patients should be made aware if anyone else is present in the room with the physician. Family practice SOO certification examinations in Canada do not currently require the use of the EHR. If the use of the EHR is incorporated into the CFPC SOO certification exams in the future, resident physicians may benefit from formal teaching on using the EHR effectively during patient appointments.

A recent study of EHR time allocation among primary care clinicians found that EHR time during patient visits varied widely between physicians, ranging from a low of 20% to over 50% [51]. For video visits, the average proportion of time spent on EHR work during the visit was 39% (95% CI 31-47%) [51]. This result is similar to the findings from this study. The median proportion of visit time spent interacting with the computer was 30% (IQR 14%-36%) for residents for the NoHx case, 30% (IQR 26%-34%) for staff physicians for the NoHx case, 38% (IQR 32%-50%) for staff physicians for the PMHx case, and 42% (IQR 32%-47%) for resident physicians for the PMHx case.

### Limitations

A major limitation of this study was that participants interpreted the instructions differently in terms of which EHR tasks were required. Some participants attempted to order laboratory tests and imaging and to arrange for referrals and follow-up appointments, while others only clicked on the “Progress Notes” section of the EHR. Although this is in part due to the exact wording of the instructions, it also reflects variation in day-to-day practice patterns regarding whether to document in the EHR while the patient is present or after the medical encounter has concluded. All participants knew that they would have an additional 5 minutes after the patient left to document in the EHR; however, many chose to document while the SP was present. One staff physician took notes using pen and paper and did not use the computer at all until after the medical encounters with each SP had ended. It is likely that each participant followed their usual practice for how much to interact with the EHR during a telemedicine appointment, versus leaving documentation until after the encounter with the patient was over. Therefore, the variability in the EHR interaction during telemedicine encounters observed in this exploratory study is a valuable finding, despite the possible variation in interpretation of instructions.

Exploratory qualitative coding of the video recordings was conducted by only one person. While this is a major limitation in terms of the qualitative findings, the coding of participants’ verbal and nonverbal behaviors was extremely time-consuming. It was not feasible to have 2 team members devote this much time to qualitative coding, given the other components of this study that required 2 scorers. While not all encounter videos were coded for verbal and nonverbal signs of stress/CL, the coding was informed by detailed viewing of all 32 videos. As discussed in the *Methods* section, the qualitative coder watched all 32 encounter videos multiple times to accurately calculate the time spent interacting with the computer, as well as EHR page/navigation counts. The signs of stress/CL were noted in all encounters to varying extents.

Technical challenges included errors in Zoom-generated transcripts due to nonlexical conversational sounds, filler words, and the inability of Zoom to determine the speaker when the physician and SP spoke at the same time. Transcripts had to be manually corrected to ensure that the correct speaker and dialogue were recorded. Some participants did not hear time prompts over Zoom, resulting in variable appointment lengths. To address this issue, active time spent using the EHR was calculated as a proportion of overall appointment time. One of the SPs displayed inconsistent acting, which affected the participants' ability to elicit the medical history and interpret nonverbal cues of the symptoms being portrayed.

Physician examiners who rated the participants for PC and overall clinical performance had prior interactions with some participants while working at the same clinic as them. This could potentially have influenced their ratings of the participants because they could not be blinded to the participants’ identities. Participants volunteered to participate in the study; thus, selection bias could have influenced the results.

### Conclusions

In this small exploratory mixed methods study, interaction with the EHR or internet searches conducted during video telemedicine appointments negatively impacted patient-centered communication and overall clinical performance. The exploratory inductive qualitative coding of the video recordings identified possible verbal and nonverbal markers of increased stress/CL due to multitasking by interacting with the computer and the SP simultaneously. Future research on the potential for automated analysis of these verbal and nonverbal markers as indicators of stress or increased CL may be useful.

A digital communication curriculum for telemedicine appointments may be beneficial to medical education. Physicians may benefit from training on remaining patient-centered and communicating effectively while using the EHR during virtual appointments. Video telemedicine communication best practices include directing eye gaze at the camera, being mindful of the length of pauses after physician statements, using clear language, and practicing active listening. Effective communication practices during telemedicine appointments can facilitate contemporaneous charting while minimizing the potential negative impacts on patients caused by physicians’ distraction from the computer. When developing SP simulation scenarios for assessing medical students and resident physicians where the use of the EHR is expected, clear instructions should be provided on which tasks involving the EHR are required.

## Supplementary material

10.2196/84916 Multimedia Appendix 1 Participant instructions.

10.2196/84916 Multimedia Appendix 2 Detailed statistical results.

## Abbreviations

CFPC: College of Family Physicians of Canada
CL: cognitive load
EHR: electronic health record
NASA-TLX: National Aeronautics and Space Administration Task Load Index
NoHx case: simulation case that did not include any past medical history in the EHR
PC: patient-centeredness
PMHx case: simulation case that included some past medical history in the EHR
PPPC-R: Revised Patient Perception of Patient-Centeredness questionnaire
SOO: simulated office oral
SP: standardized patient
