# Supplementary material for: The Impact of Electronic Health Records on Family Physicians During Simulated Virtual Encounters: Exploratory Mixed Methods Study
Source: JMIR Med Inform. 2026 May 19;14:e84916. doi: 10.2196/84916 (PMC13186519; doi:10.2196/84916)
Supplement: Multimedia Appendix 1 [file medinform-v14-e84916-s001.pdf]

Multimedia Appendix 1: Participant Instructions

This is a SIMULATED patient encounter, using a trained actor to reflect a realistic, routine family medicine office visit for a **patient new to you**. You will be expected to completely document the encounter, using our electronic health record. You will have about 20 minutes to interact with the patient and an additional 5 minutes to complete your documentation of the encounter.

Our research team asks that both the participant and simulated patient role-play for the entire session. The standardized patient will tell you when there are 3-minutes remaining in the 20-minute visit. Please plan to finish your encounter at that time, to allow 5-minutes for the charting of the encounter in the electronic health record. To expedite this, at the 20 minute point, the encounter will be interrupted and patient will leave. **The total time scheduled for this Standardized Patient-Participant encounter is 25-minutes.**

Encounters will occur in the Epic Sandbox environment. Personal EHR customizations, template, and smart phrases will not carry over to the Sandbox environment.

The standardized patients will clearly understand that physical examination would not occur with a virtual visit (and as well, they will have already provided their consent for a virtual visit, so there is no requirement to repeat this request for consent at the beginning of the visit. Should an examination be recommended as an outcome of the visit, you should inform the patient about the need to make a face to face future appointment.

Though new to your practice, the standardized patient will be found in the electronic health record with at least an appointment today. Simulated historical information may be available in the electronic health record for each patient.

Finally, we would ask that after completion of your session, you do not discuss the cases with your colleagues so, should they also participate in the study, they will do so without any prior knowledge of what to expect.
