# Supplementary material for: The Impact of Electronic Health Records on Family Physicians During Simulated Virtual Encounters: Exploratory Mixed Methods Study
Source: JMIR Med Inform. 2026 May 19;14:e84916. doi: 10.2196/84916 (PMC13186519; doi:10.2196/84916)
Supplement: Multimedia Appendix 2 [file medinform-v14-e84916-s002.pdf]

Multimedia Appendix 2: Detailed Statistical Results

Table S1. Median (IQR) values and Wilcoxon-Mann-Whitney (WMW) rank sum test results by physician training level for the past medical history (*PMHx)* case (N=16).

Variables	Residents *PMHx* case (n=10)	Staff *PMHx* case (n=6)	*P* value	Holm-Bonferroni Threshold[a]
**Computer use**				
Computer use time proportion	0.42 (0.32-0.47)	0.38 (0.32-0.50)	.87	.02
Navigation count	9.5 (4.2-26.0)	11.0 (2.5-20.2)	.45	.008
Page count	3.0 (2.00-5.00)	2.5 (1.25-3.75)	.51	.01
**Physician performance**				
Patient-centeredness score	6.0 (4.0-8.0)	12.5 (9.0-16.0)	.03	.006
SOO[b] exam total score	20.0 (13.0-24.8)	27.0 (18.5-34.8)	.14	.006
PPPC-R[c]	3.2 (2.9-3.5)	1.8 (1.7-2.4)	.01	.005
**Cognitive load (NASA-TLX[d])**				
Raw NASA-TLX mean	46.3 (40.2-53.0	44.2 (36.5-53.5)	.87	.02
NASA frustration	54.5 (21.5-70.5)	44.0 (12.2-60.0)	.51	.01
NASA mental demand	65.0 (50.8-74.0)	69.0 (53.5-79.2)	.79	.02
NASA time pressure	37.5 (31.2-67.2)	49.0 (46.5-66.5)	.36	.007

[a]Holm-Bonferroni correction method: threshold = α / (*m*-*i* + 1) where *i = rank, m= total tests*.
[b]SOO: simulated office oral.
[c]PPPC-R: revised patient perception of patient-centeredness (PPPC) questionnaire. On the PPPC-R scale, a lower score indicates better performance due to reverse anchoring.
[d]NASA-TLX: National Aeronautics and Space Administration Task Load Index.

Table S2. Median (IQR) values and Wilcoxon-Mann-Whitney (WMW) rank sum test results by physician training level for the no history (*NoHx*) case (N=16).

Variable	Residents *NoHx* case (n=10)	Staff *NoHx* case (n=6)	*P* value	Holm-Bonferroni Threshold[a]
**Computer use**				
Computer use time proportion	0.30 (0.14-0.36)	0.30 (0.26-0.34)	>.99	.02
Navigation count	1.0 (1.0-3.5)	1.5 (1.0-6.5)	>.99	.02
Page count	1.0 (1.0-2.0)	1.5 (1.0-2.8)	>.99	.02
**Physician performance**				
Patient-centeredness score	16.5 (12.0-17.8)	21.0 (18.5-22.8)	.02	.006
SOO[b] exam total score	32.0 (27.0-34.5)	44.0 (41.5-46.5)	.008	.005
PPPC-R score [c]	1.8 (1.6-2.4)	1.4 (1.2-1.8)	.17	.006
**Cognitive load (NASA-TLX[d])**				
Raw NASA-TLX mean	54.0 (47.2-57.2)	39.9 (32.7-50.9)	.26	.007
NASA frustration	54.5 (38.8-69.2)	15.5 (8.5-52.5)	.33	.008
NASA mental demand	72.5 (60.2-78.8)	64.5 (39.5-81.2)	.74	.01
NASA time pressure	55.5 (19.0-76.0)	57.5 (55.0-60.0)	.96	.01

[a]Holm-Bonferroni correction method: threshold = α / (*m*-*i* + 1) where *i = rank, m= total tests*.
[b]SOO: simulated office oral.
[c]PPPC-R: revised patient perception of patient-centeredness (PPPC) questionnaire. On the PPPC-R scale, a lower score indicates better performance due to reverse anchoring.
[d]NASA-TLX: National Aeronautics and Space Administration Task Load Index.

Table S3. Kendall's tau-b (τb) correlation results for resident physicians for the past medical history (*PMHx*) case (n=10).

Variable	PC[a] score	SOO[b] total score	PPPC-R[c]	Page count	Navigation count	NASA MD[d]	NASA TP[e]	NASA FR[f]	NASA 6 domain mean[g]
**Computer use**									
τ_b	-0.35	-0.25	-0.16	0.35	0.25	0.64	0.31	-0.11	0.18
P value	.17	.32	.53	.17	.32	.009	.21	.65	.47
Holm-Bonferroni Threshold[h]	.006	.01	.02	.006	.01	.006	.008	.05	.02

[a]PC: patient-centeredness
[b]SOO: simulated office oral exam
[c]PPPC-R: revised patient perception of patient-centeredness (PPPC) questionnaire. On the PPPC-R scale, a lower score indicates better performance due to reverse anchoring. Directions of correlation reflect the original outputs in RStudio.
[d]NASA MD: mental demand domain of the National Aeronautics and Space Administration Task Load Index
[e]NASA TP: time pressure domain of the National Aeronautics and Space Administration Task Load Index
[f]NASA FR: frustration domain of the National Aeronautics and Space Administration Task Load Index
[g]NASA 6 domain mean: mean of the 6 domains of the raw National Aeronautics and Space Administration Task Load Index
[h]Holm-Bonferroni correction method: threshold = $\alpha / (m-i + 1)$ where *i = rank, m= total tests*

Table S4. Kendall's tau-b (τb) correlation results for staff physicians for the past medical history (*PMHx*) case (n=6).

Variable	PC[a] score	SOO[b] total score	PPPC-R[c]	Page count	Navigation count	NASA MD[d]	NASA TP[e]	NASA FR[f]	NASA 6 domain mean[g]
**Computer use**									
τ_b	-0.47	-0.55	0.97	0.87	0.60	0.14	0.47	0.41	0.47
P value	.27	.13	.007	.02	.14	.70	.27	.25	.27
Holm-Bonferroni Threshold[h]	.01	.007	.006	.006	.008	.05	.01	.01	.01

[a]PC: patient-centeredness
[b]SOO: simulated office oral exam
[c]PPPC-R: revised patient perception of patient-centeredness (PPPC) questionnaire. On the PPPC-R scale, a lower score indicates better performance due to reverse anchoring. Directions of correlation reflect the original outputs in RStudio.
[d]NASA MD: mental demand domain of the National Aeronautics and Space Administration Task Load Index
[e]NASA TP: time pressure domain of the National Aeronautics and Space Administration Task Load Index
[f]NASA FR: frustration domain of the National Aeronautics and Space Administration Task Load Index
[g]NASA 6 domain mean: mean of the 6 domains of the raw National Aeronautics and Space Administration Task Load Index
[h]Holm-Bonferroni correction method: threshold = $\alpha / (m-i + 1)$ where *i = rank, m= total tests*

Table S5. Kendall's tau-b (τb) correlation results for resident physicians for the no history (*NoHx*) case (n=10).

Variable	PC[a] score	SOO[b] total score	PPPC-R[c]	Page count	Navigation count	NASA MD[d]	NASA TP[e]	NASA FR[f]	NASA 6 domain mean[g]
**Computer use**									
τ_b	-0.20	-0.04	0.11	0.36	0.38	0.31	0.60	0.04	0.33
P value	.42	.86	.73	.18	.15	.21	.02	.86	.22
Holm-Bonferroni Thresholdh	.01	.02	.02	.007	.006	.008	.006	.02	.01

[a]PC: patient-centeredness
[b]SOO: simulated office oral exam
[c]PPPC-R: revised patient perception of patient-centeredness (PPPC) questionnaire. On the PPPC-R scale, a lower score indicates better performance due to reverse anchoring. Directions of correlation reflect the original outputs in RStudio.
[d]NASA MD: mental demand domain of the National Aeronautics and Space Administration Task Load Index
[e]NASA TP: time pressure domain of the National Aeronautics and Space Administration Task Load Index
[f]NASA FR: frustration domain of the National Aeronautics and Space Administration Task Load Index
[g]NASA 6 domain mean: mean of the 6 domains of the raw National Aeronautics and Space Administration Task Load Index
[h]Holm-Bonferroni correction method: threshold = $\alpha / (m-i + 1)$ where *i = rank, m= total tests*

Table S6. Kendall's tau-b (τb) correlation results for staff physicians for the no history (*NoHx*) case (n=6).

Variable	PC[a] score	SOO[b] total score	PPPC -R[c]	Page count	Navigation count	NASA MD[d]	NASA TP[e]	NASA FRf	NASA 6 domain mean[g]
**Computer use**									
τ_b	0.14	-0.47	-0.41	0.69	0.69	-0.47	-0.21	-0 .20	-0.73
P value	.70	.27	.25	.06	.06	.27	.56	.72	.06
Holm-Bonferroni Threshold[h]	.02	.01	.008	.006	.006	.01	.02	.05	.006

[a]PC: patient-centeredness
[b]SOO: simulated office oral exam
[c]PPPC-R: revised patient perception of patient-centeredness (PPPC) questionnaire. On the PPPC-R scale, a lower score indicates better performance due to reverse anchoring. Directions of correlation reflect the original outputs in RStudio.
[d]NASA MD: mental demand domain of the National Aeronautics and Space Administration Task Load Index
[e]NASA TP: time pressure domain of the National Aeronautics and Space Administration Task Load Index
[f]NASA FR: frustration domain of the National Aeronautics and Space Administration Task Load Index
[g]NASA 6 domain mean: mean of the 6 domains of the raw National Aeronautics and Space Administration Task Load Index
[h]Holm-Bonferroni correction method: threshold = $\alpha / (m-i + 1)$ where *i = rank, m= total tests*
